# Transient Leukemia in an Adult Dog With Cutaneous T‐Cell Lymphoma

**DOI:** 10.1111/vcp.70025

**Published:** 2025-06-16

**Authors:** Raquel Moreira, Malgorzata Ossowska, Oriol Jornet‐Rius, Marta Santos, Francesco Cian

**Affiliations:** ^1^ Batt Laboratories Coventry UK; ^2^ The Royal (Dick) School of Veterinary Studies and the Roslin Institute, University of Edinburgh Edinburgh UK; ^3^ Blaise Referral Hospital Birmingham UK; ^4^ Zoetis Spain S.L.U. Madrid Spain; ^5^ Cytology and Hematology Diagnostic Services, Laboratory of Histology and Embryology, School of Medicine and Biomedical Sciences, ICBAS—UP University of Porto Porto Portugal

**Keywords:** canine, flow cytometry, immunocytochemistry, immunohistochemistry, marked leukocytosis, non‐epitheliotropic lymphoma

## Abstract

A 5‐year‐old neutered female Beagle was presented with lethargy, vomiting, and reduced appetite. Physical examination revealed mild hyperthermia and a cutaneous thickness on the right thigh. Hematology showed marked leukocytosis and moderate thrombocytopenia, consisting of 80% of atypical circulating cells, initially suggesting acute undifferentiated leukemia. Two weeks later, repeated hematology revealed unremarkable results. Cytology of the skin lesion showed a round cell neoplasia with features similar to the atypical circulating cells. Following the development of multiple cutaneous nodules and recurrence of clinical signs, further diagnostics, including flow cytometry, histopathology, and immunohistochemistry (IHC) of the skin nodules, as well as PCR for antigen receptor rearrangement (PARR) and immunocytochemistry (ICC) from the initial blood smear, confirmed a neoplastic T‐cell proliferation consistent with cutaneous T‐cell lymphoma with a probable transient leukemic phase. Despite chemotherapy, remission was short‐lived, and the patient relapsed, ultimately leading to euthanasia. This case highlights a rare instance of transient leukemia likely originating from a primary cutaneous T‐cell lymphoma, emphasizing the need for comprehensive diagnostic workups, combining hematology, biochemistry, cytology, flow cytometry, and immunophenotyping to avoid misclassification in hematologic malignancies.

## 1 Case Presentation

A 5‐year‐old neutered female Beagle presented to its primary veterinarian with clinical signs of lethargy, vomiting, and decreased appetite. Mild hyperthermia (39.7°C [37.5°C–39.3°C]) and a recently noted cutaneous thickening on the right thigh were seen on physical exam, with no other abnormalities noted.

**FIGURE 1:**
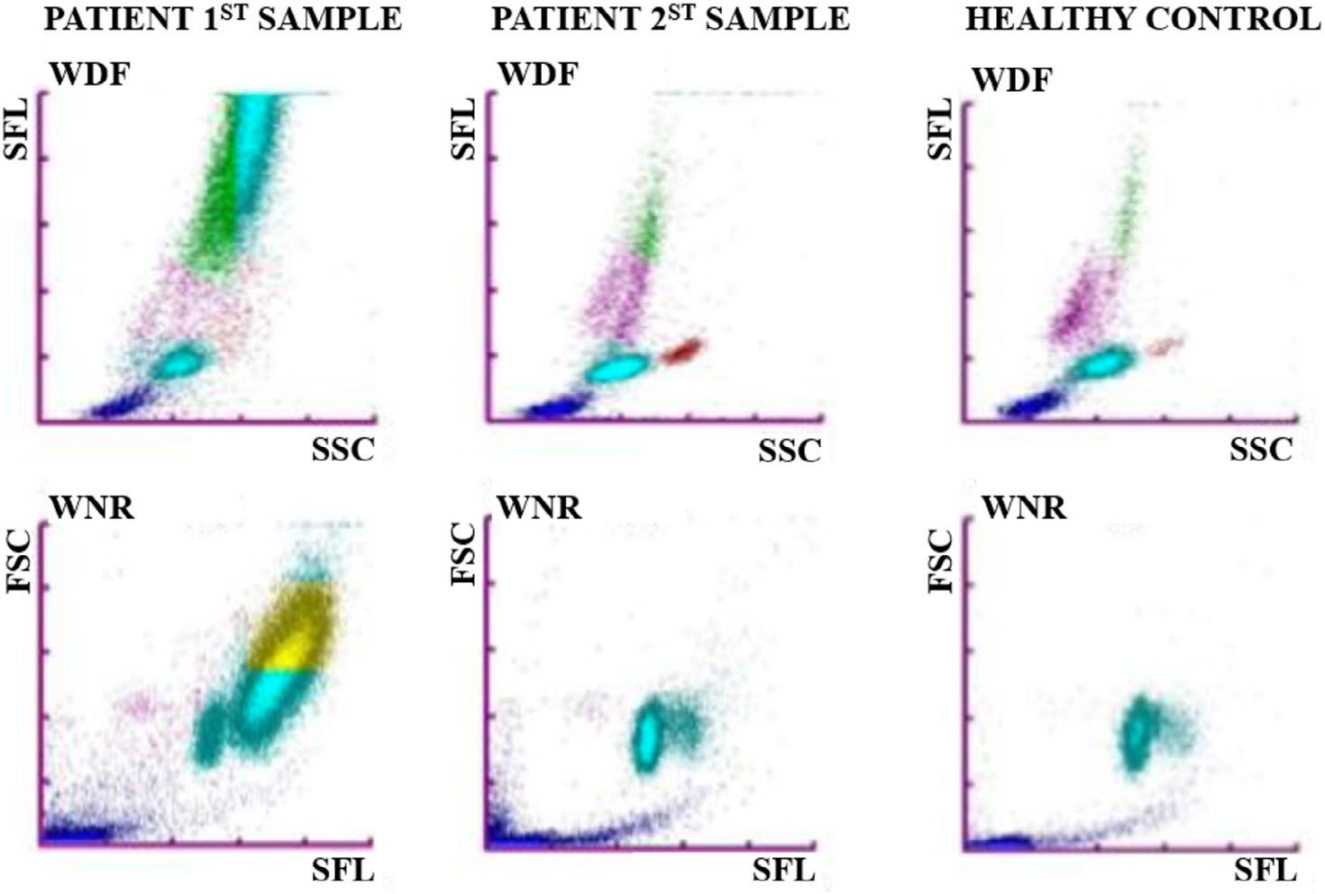
Sysmex XN‐V analyzer scattergrams of the WBC differential fluorescence (WDF), and white count/nucleated red blood cells channel (WNR). Patient's first sample—WDF Scattergram: Initial hematology result showing an atypical cellular distribution with a prominent population of highly fluorescent cells. The WNR scattergram indicates a large population of WBCs (light blue) and a significant group of cells in the basophil area (yellow). Patient's second sample—WDF Scattergram: Subsequent hematology sample taken two weeks later, demonstrating a normal distribution of leukocyte subpopulations. Healthy canine control—WDF Scattergram: Hematology sample from a canine patient with values within the normal reference range.

**FIGURE 2:**
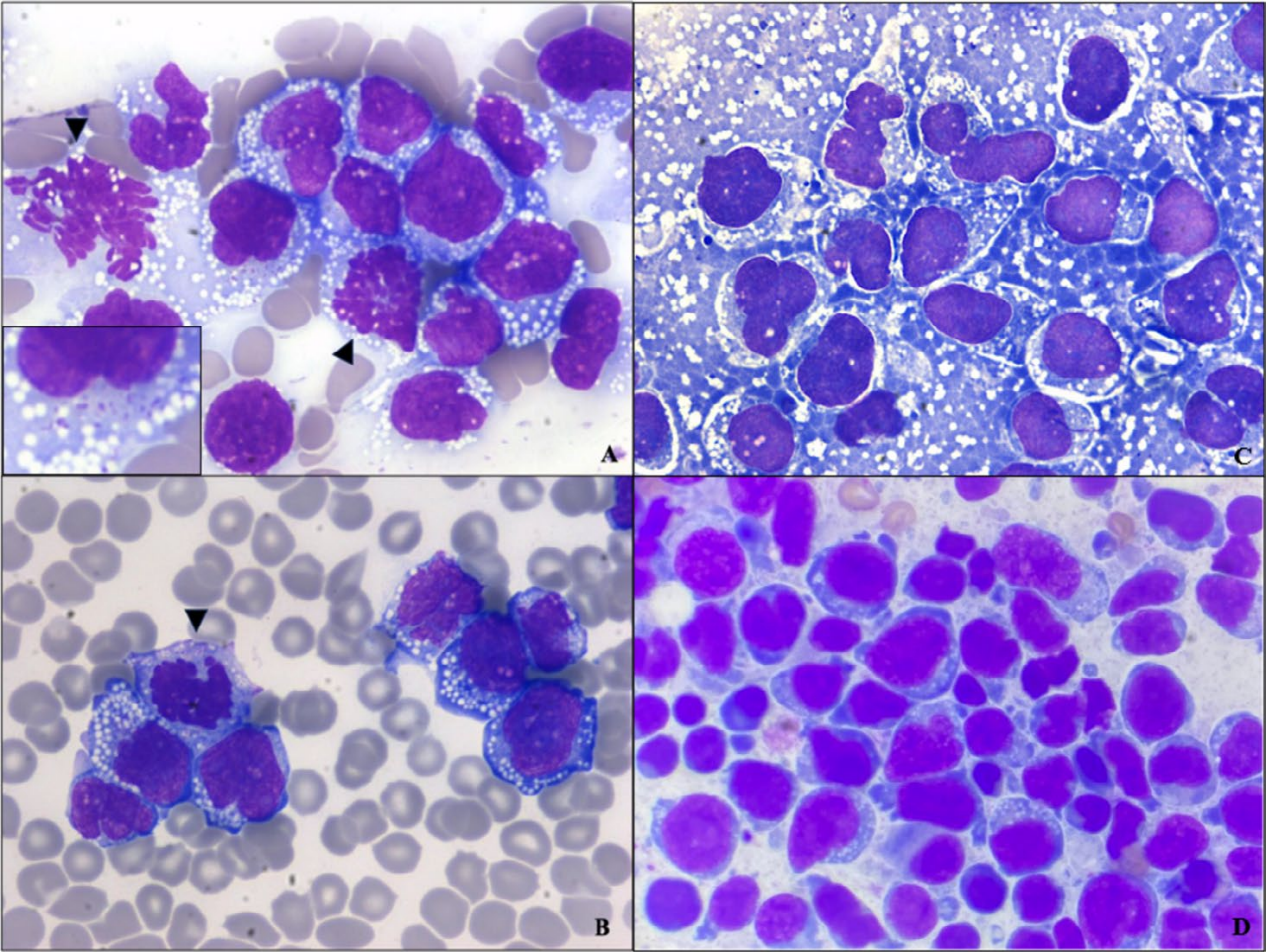
Cytologic evaluation of atypical hematopoietic and neoplastic cells. Blood smear from the first hematology sampling reveals circulating atypical hematopoietic cells at the feathered edge (A) and monolayer (B), with an inset in A enhancing the visualization of the fine pink cytoplasmic granules. Mitotic figure indicated by arrowhead. Fine needle aspiration of the right lateral thigh cutaneous nodule (C) shows the predominant neoplastic cell population. Aspiration of the right prescapular lymph node (D) reveals a predominance of an undetermined round cell population. Wright‐Giemsa, ×50 objective (A–C); Hemacolor, ×100 (Oil immersion) objective (D).

Biochemistry evaluation revealed a marked C‐reactive protein (CRP) elevation (201 mg/L [0–15 mg/L]) with no other significant abnormalities. Hematology results (Sysmex XN‐1000 V hematology analyzer, Sysmex Corporation, Norderstedt, Germany) indicated moderate thrombocytopenia (62 × 10^9^/L [150–500 × 10^9^/L]) and marked leukocytosis (46.09 × 10^9^/L) (Table 1), with abnormal scattergram analysis suggesting atypical cell distribution (Figure 1). A blood smear was also examined, revealing a high number of abnormal round cells, accounting for 80% of all nucleated cells and displaying distinct morphological characteristics, with evidence of leukergy (Figure 2A,B). These cells measured 10–25 μm in size and had a moderate amount of deeply basophilic cytoplasm, containing frequent small clear vacuoles and occasional fine pink cytoplasmic granules. The nuclei were eccentrically positioned, round to cerebriform in shape, with finely stippled chromatin, one to multiple prominent nucleoli, and evidence of mitotic activity. Rare binucleated cells were also noted. A manual platelet count confirmed thrombocytopenia, with no platelet clumping observed. Based on these findings, acute undifferentiated leukemia was suspected. Flow cytometry analysis was recommended, but could not be performed as, due to extended transportation time, the sample was not viable for analysis. Consequently, a repeat hematology assessment using the same analyzer was performed 2 weeks later. The new sample showed an unremarkable leukogram and erythrogram, with a shift toward mild thrombocytosis (650 × 10^9^/L [150–500 × 10^9^/L]), and no atypical circulating cells on the blood smear (Table 1).

**TABLE 1:** Presentation of the hematology results obtained using a Sysmex XN‐V hematology analyzer.

	Results (1)	Results (1*)	Results (2)	RI	Units
Erythrogram					
RBC	6.33		6.81	5.50–8.50	10^12^/L
HGB	14.7		16.2	12.0–18.0	g/dL
HCT	0.43		0.47	0.37–0.55	Ratio
MCV	67.3		69.2	60.0–77.0	fL
MCH	23.1		23.8	19.0–26.0	Pg
MCHC	34.3		34.4	32.0–39.0	g/dL
RDW	13.6		16.7		%
Platelets	**↓ 62**		**↑ 650**	150–500	10^9^/L
Leukogram					
Leukocytes	**↑ 46.1**	**↑ 46.1**	11.3	6.0–15.0	10^9^/L
Neutrophils	**↑ 20.95**	7.84	8.56	3.00–11.50	10^9^/L
% Neutrophils	45.5	17	75.9		%
Lymphocytes	**↓ 0.27**	**↓ 0.46**	1.30	1.00–5.00	10^9^/L
% Lymphocytes	0.6	1	11.5		%
Monocytes	**↑ 4.86**	0.92	0.80	0.00–1.40	10^9^/L
% Monocytes	10.5	2	7.1		%
Eosinophils	0.33	0	0.57	0.00–1.25	10^9^/L
% Eosinophils	0.7	0	5.1		%
Basophils	**↑ 19.68**	0	0.05	0.00–0.0.20	10^9^/L
% Basophils	42.7	0	0.4		%
NRBC	0.41	0	0.06		10^9^/L
% NRBC	0.9	0	0.5		%
Atypical cells		**↑ 36.87**		0.00	10^9^/L
% Atypical cells		**↑ 80%**		0.00	%

*Note:* Results (1) = First hematology results; Results (1*) = First hematology results—manual leukocyte differential count; Results (2) = Second hematology results (two weeks later); RI = Reference intervals. Bold values are statistically significant.

Simultaneously, a cytology sample was collected from the cutaneous thickening on the right thigh, which had developed into a nodular lesion. Moderate numbers of round cells were present that were cytomorphologically similar to those seen in the blood (Figure 2C). These findings were consistent with an undetermined round cell neoplasia, most likely lymphoid, mirroring the morphological features observed in the blood smear of the presentation.

**FIGURE 3:**
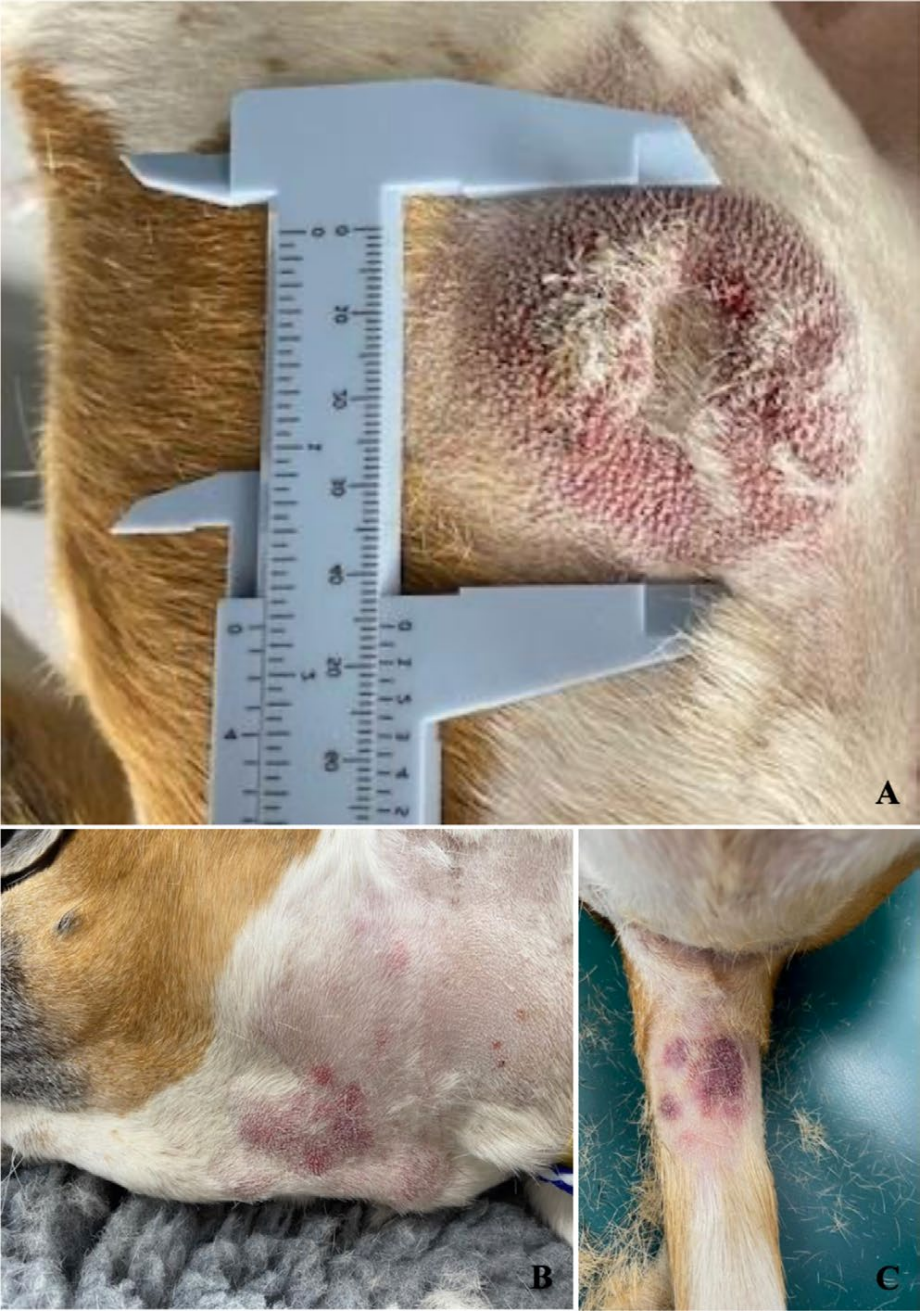
Macroscopic appearance of the right lateral thigh skin nodule (A) and multiple firm, erythematous subcutaneous nodules observed across various body regions at the time of presentation to the referral hospital (B, C).

The following day (15 days after the initial presentation), the patient was referred to a specialized hospital for evaluation by an oncologist due to the development of multiple firm erythematous subcutaneous nodules across various body regions (Figure 3), along with lethargy and mild hyporexia. Upon examination by the oncologist at the referral hospital, moderate lymphadenomegaly was noted in the mandibular and right prescapular lymph nodes. In‐house cytology (Figure 2D) revealed a large population of round cells, displaying features similar to those observed in both the peripheral blood and the cutaneous mass, though slightly less vacuolated. Some small lymphocytes, a few plasma cells, and neutrophils were also observed. These features led the referral hospital to suspect a large cell lymphoma. Based on this presumptive diagnosis, the oncologist initiated chemotherapy, implementing an alternating sequential protocol with cyclophosphamide, doxorubicin, vincristine, and prednisolone (CHOP) while awaiting additional test results. The clinical staging, including thoracic radiographs and an abdominal ultrasound with liver and spleen cytology, revealed no significant abnormalities.

**FIGURE 4:**
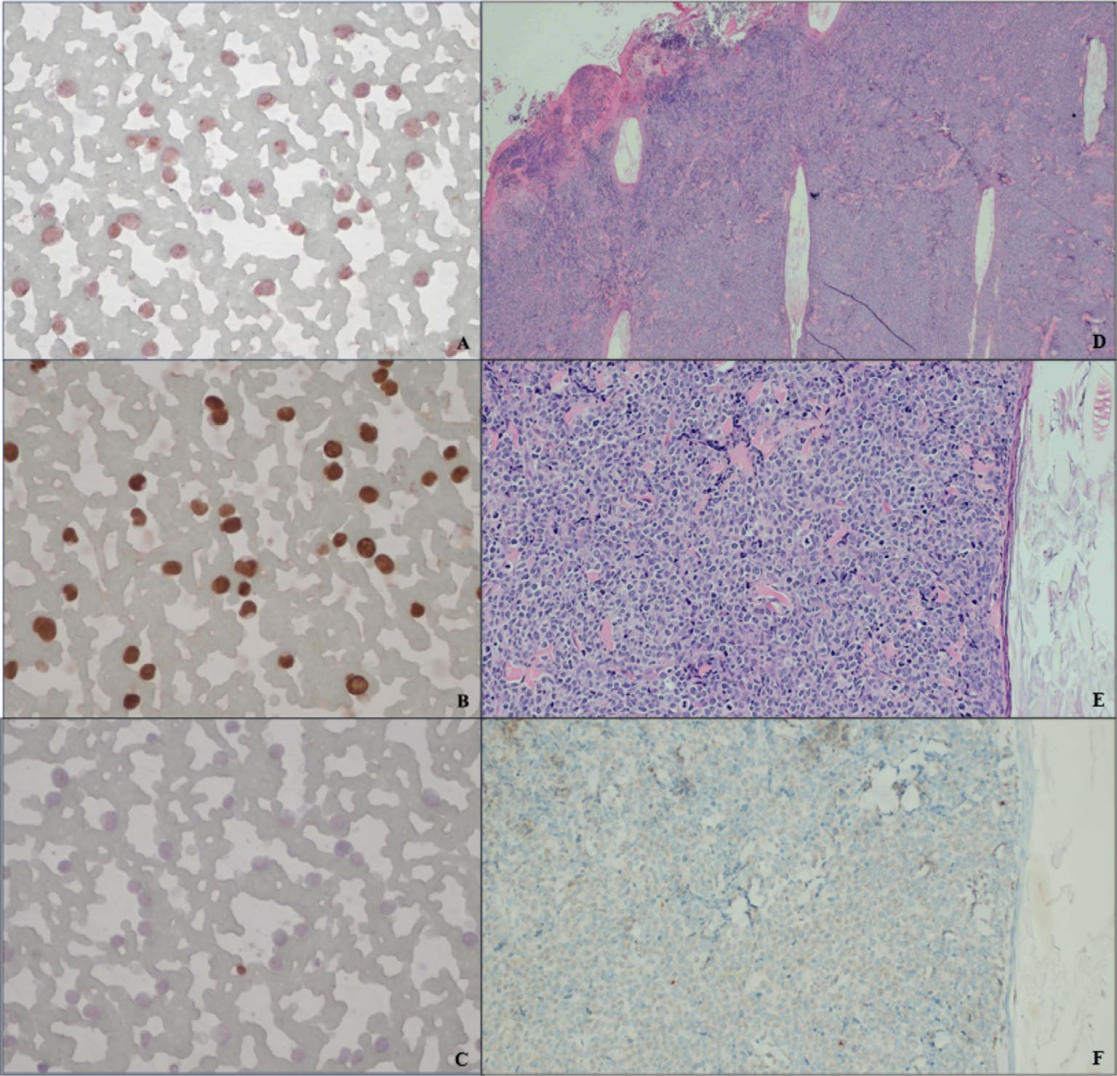
Immunocytochemistry results from the blood smear, showing over 80% of the atypical population displaying faint cytoplasmic/membrane CD3 staining (A) and more than 95% exhibiting positive Ki67 expression (B). Pax5 marker expression is negative in the atypical cells, with only rare small lymphocytes showing positive staining (C). Histopathology from the right lateral thigh cutaneous nodule shows a packed neoplastic population of round cells without evidence of epitheliotropism (D) and surrounding a hair follicle (E). Immunohistochemistry results on a section of the same nodule (F) reveal variable and predominantly faint CD3 staining. H&E, ×4 and ×20 objectives (D, E); Diaminobenzidine chromogen with hematoxylin counterstain, ×40 objective (A–C), ×20 objective (F).

Both the PARR test and ICC were performed on previously stained blood smears from the initial hematology samples containing the atypical cells. The PARR test detected a weak monoclonal proliferation of T‐lymphocytes, suggestive of T‐cell lymphoma/leukemia. ICC revealed that over 80% of the target population showed faint cytoplasmic/membrane for CD3 staining (Figure 4A), more than 95% positivity for Ki67 expression (Figure 4B), and negative Pax5 staining in the neoplastic cells (Figure 4C), supporting the previous diagnosis.

Flow cytometry was conducted on fine‐needle aspiration rinse fluids of the first observed nodule, located on the right lateral thigh, as well as on a recently developed nodule on the right ear. The results were compatible with CD8+ T‐cell lymphoma/leukemia (Table 2). Additionally, an incisional biopsy of the right lateral thigh nodule was performed. Histopathology revealed densely packed round cells of medium to large size and showed moderate nuclear pleomorphism. The nuclear features were highly variable, with a predominance of small nucleoli and a high number of mitotic figures (up to 20/hpf) (Figure 4D,E). Although there was no convincing evidence of epitheliotropism, the neoplastic cells extended into the underlying fat. A diagnosis of cutaneous non‐epitheliotropic lymphoma was made. IHC for CD3 (Figure 4F) demonstrated variable and faint staining in most neoplastic cells, consistent with T‐cell origin.

**TABLE 2:** Presentation of the flow cytometry findings on the mass located on the leg and ear.

Markers	Leg mass	Ear mass
CD5	Positive in 52% of TP	Positive in 95% of TP
CD21	Positive in 17% of TP	Positive in 13% of TP
CD8	NT	Positive in 46% of TP
CD4	NT	Positive in 16% of TP
Notes	Very low cellularity	Low cellularity

Abbreviations: NT, not tested due to scarce cellularity; TP, target population, corresponding to the region of intermediate and large lymphoid/mononuclear cells.

One week after starting the chemotherapy, the patient showed clinical improvement and a partial response, with lymph node sizes returning to normal and a decrease in the size of cutaneous lesions. However, following the identification of a large T‐cell lymphoma in the additional results, the treatment protocol was altered to a modified COEP (cyclophosphamide, epirubicin, prednisolone, and vincristine), incorporating lomustine [1]. Remarkably, the patient achieved complete remission after the first lomustine treatment. Unfortunately, the remission was short‐term (22 days), as multiple new cutaneous lesions, cytologically identical to the initial nodule, emerged primarily on the head and ventral abdomen. Notably, lymph node sizes remained normal, and leukocyte counts were within normal limits, with no atypical circulating cells observed on blood smear examination performed during hematology analysis. Throughout the chemotherapy protocol, all hematology analyses showed no signs of atypical cells in circulation. Despite a rescue treatment attempted with asparaginase, which showed no response, and subsequent administration of doxorubicin, the patient developed progressive disease and neurologic signs. Rescue treatment with cytarabine infusion was attempted, but no response was achieved, and neurologic signs progressed. Ultimately, due to the progressive nature of the disease and deteriorating quality of life, the owner elected euthanasia, and no necropsy was performed.

## 2 Discussion

In this report, we describe a case of a five‐year‐old dog with a marked leukocytosis and large, abnormal, round cells observed on blood smear at the clinical presentation, which prompted an immediate, comprehensive investigation. In cases of marked leukocytosis, scattergram analysis from the Sysmex XN‐1000 V analyzer can show discrepancies in cell count compared to manual leukocyte differential counts. This phenomenon has been previously reported in cases of acute lymphoid leukemia using the same hematology analyzer [2]. In the present case, abnormal cells were classified within basophil, neutrophil, and monocyte categories on manual differential counts, but interestingly, these cells were concentrated within the basophilic region on the scattergram. This can be likely influenced by their large size, cytoplasmic vacuoles, and fine cytoplasmic granules, although the specific impact of vacuoles on fluorescence‐based differentiation in Sysmex analyzers has not been well documented in the literature. Although flow cytometry was not performed on the first blood sample, a presumptive diagnosis of acute undifferentiated leukemia (AUL) was made, given the presence of 80% blast cells in circulation. According to the literature, the threshold for considering leukemia is ≥ 20% blast cells, further supporting the diagnosis in this case [3, 4]. Two weeks later, the cytologic examination of the right lateral thigh nodule revealed features consistent with round cell neoplasia. The identical appearance of cells in this nodule and the initial blood smear suggested a common origin. Further testing, including PARR, ICC, and IHC, provided evidence that the atypical cells in both the blood and the cutaneous lesion were of a monoclonal neoplastic T‐cell origin, supporting a diagnosis of T‐cell cutaneous lymphoma with leukemic dissemination at the first presentation. A marked increase in CRP is indicative of an underlying inflammatory process [5]. Although nonspecific, CRP is highly sensitive and has also been reported in association with lymphatic neoplasms [5, 6].

The peculiar aspect of this case was the disappearance of circulating atypical cells in follow‐up hematology 2 weeks after the initial presentation, despite no chemotherapy or glucocorticoid treatment had been initiated. Several hypotheses were considered, including potential sample mishandling, which was ruled out after verifying patient identity and consistency in RBC parameters between samples. Additionally, percutaneous aspiration of cutaneous/subcutaneous neoplastic cells during blood collection was dismissed, as the sample was taken from the jugular vein and no cervical masses were present. It could also be hypothesized that the circulating neoplastic cells presented in the blood at the time of presentation were removed by an immune‐mediated process. While immune‐mediated clearance of neoplastic cells is a possibility, complete elimination is unlikely given the clinical progression of the case. The development of multiple cutaneous lesions and lymph node involvement suggests that neoplastic cells transiently entered the bloodstream. This hematologic dissemination likely contributed to the subsequent emergence of additional cutaneous nodules.

Although it has not yet been described in veterinary medicine, transient leukemia has been occasionally reported in human medicine, particularly in cases of acute lymphoblastic leukemia (ALL) [7, 8, 9]. The underlying mechanisms remain scarcely understood; however, the immune system is thought to play a crucial role in these cases. Spontaneous and transient remission in ALL, in human medicine, is thought to be driven by immune responses and has been documented in association with fever, sepsis, or acute stress [8]. Previous studies have reported a variable remission time ranging from 5 days to 7 weeks, with almost all cases experiencing a relapse with a blast phenotype similar to the one reported at presentation [8, 9]. In this case, the dog exhibited mild hyperthermia during the initial clinical presentation, and no medical treatment was prescribed, suggesting the potential involvement of the immune system. However, no relapse of leukemic cells was observed, possibly due to early chemotherapy intervention or the short duration of the patient's clinical course.

The lack of bone marrow aspiration and core biopsy is a limitation, as it prevents definitive confirmation or exclusion of bone marrow involvement. While some literature underscores the importance of this evaluation, others note that circulating neoplastic cells do not necessarily indicate marrow infiltration [10, 11, 12, 13]. The transient thrombocytopenia at presentation and rapid shift from a leukemic to aleukemic state without treatment make acute leukemia less likely, as it typically presents with persistent cytopenias [3, 14]. However, bone marrow involvement cannot be ruled out. Graff et al. identified thrombocytopenia and > 10% neoplastic lymphocytes on blood smears as predictors of marrow infiltration [10]. In this case, only moderate thrombocytopenia was initially noted, followed by mild thrombocytosis, along with a transient presence of 80% circulating neoplastic cells, further complicating interpretation. While the absence of progressive cytopenias argues against extensive marrow disease [3, 14], without direct evaluation, this remains uncertain.

Another consideration was the possibility of Sézary syndrome, a cutaneous T‐cell lymphoma with concurrent peripheral blood involvement [15]. However, the lack of epitheliotropism in this case and the transient nature of circulating neoplastic cells argue against that diagnosis [15].

Furthermore, the ICC, IHC, and PARR results revealed weak monoclonal proliferation and faint CD3 staining, potentially indicating loss of normal T‐cell receptors in the neoplastic cells.

Canine cutaneous lymphoma is classified into epitheliotropic and non‐epitheliotropic subtypes, with the latter being rare in both humans and dogs [16]. Non‐epitheliotropic lymphoma can present as cutaneous/subcutaneous nodules, as observed in this case, or plaques. While canine epitheliotropic lymphoma is almost always of T‐cell origin, the non‐epitheliotropic form can be both T‐ and B‐cell origin. Further subclassification according to the WHO criteria is uncommonly performed in canine T‐cell non‐epitheliotropic lymphoma [6]. Based on available data, prognosis for non‐epitheliotropic cutaneous lymphoma is generally guarded, with reported median survival times ranging from 1 to 36 months [17, 18]. Given the rarity of this condition and the limited information available on the treatment, the patient was treated with a modified COEP protocol adapted for use in this case of high‐grade T‐cell lymphoma [1]. Despite an initial positive response, with maximum remission achieved within 22 days, relapse occurred 36 days later, leading to an overall survival of 55 days.

In summary, to the authors' knowledge, this is the first documented case of transient acute T‐cell lymphoid leukemia likely originating from an underlying primary cutaneous T‐cell lymphoma in a dog, with cytomorphologic features suggestive of possible large granular T‐cell lymphoma. The rarity of such cases documented may be due to the difficulty in identifying transient leukemia, which is compounded by its undefined duration, the absence of specific associated signs, and the necessity of integrating hematologic findings with blood smears to detect atypical circulating cells. This case underscores the importance of combining manual and automated diagnostic methods to avoid misclassification, particularly in hematologic malignancies where atypical cells can be challenging to identify. Moreover, immunophenotyping played a pivotal role in distinguishing these neoplastic cells from other potential diagnoses, as relying solely on cytologic features can be misleading. In this case, the cells displayed large size, variable nuclear contours, delicate chromatin patterns, cytoplasmic vacuoles, and mild granulation—features that could have easily led to misdiagnosis as acute myeloid leukemia (AML) [3]. Ultimately, this case highlights the necessity of comprehensive diagnostic evaluations in cases of leukocytosis with abnormal circulating cells.

## Conflicts of Interest

The authors declare no conflicts of interest.
